# Measurement comparison and Monte Carlo analysis for volumetric‐modulated arc therapy (VMAT) delivery verification using the ArcCHECK dosimetry system

**DOI:** 10.1120/jacmp.v14i2.3929

**Published:** 2013-05-06

**Authors:** Mu‐Han Lin, Sion Koren, Iavor Veltchev, Jinsheng Li, Lu Wang, Robert A. Price, C.‐M. Ma

**Affiliations:** ^1^ Fox Chase Cancer Center Philadelphia PA USA

**Keywords:** volumetric‐modulated arc therapy (VMAT), diode array, ArcCHECK, quality assurance, Monte Carlo

## Abstract

The objective of this study is to validate the capabilities of a cylindrical diode array system for volumetric‐modulated arc therapy (VMAT) treatment quality assurance (QA). The VMAT plans were generated by the Eclipse treatment planning system (TPS) with the analytical anisotropic algorithm (AAA) for dose calculation. An in‐house Monte Carlo (MC) code was utilized as a validation tool for the TPS calculations and the ArcCHECK measurements. The megavoltage computed tomography (MVCT) of the ArcCHECK system was adopted for the geometry reconstruction in the TPS and for MC simulations. A $10\times 10\;{\text{cm}}^{2}$ open field validation was performed for both the 6 and 10 MV photon beams to validate the absolute dose calibration of the ArcCHECK system and also the TPS dose calculations for this system. The impact of the angular dependency on noncoplanar deliveries was investigated with a series of $10\times 10\;{\text{cm}}^{2}$ fields delivered with couch rotation 0° to 40°. The sensitivity of detecting the translational (1 to 10 mm) and the rotational (1° to 3°) misalignments was tested with a breast VMAT case. Ten VMAT plans (six prostate, H&N, pelvis, liver, and breast) were investigated to evaluate the agreement of the target dose and the peripheral dose among ArcCHECK measurements, and TPS and MC dose calculations. A customized acrylic plug holding an ion chamber was used to measure the dose at the center of the ArcCHECK phantom. Both the entrance and the exit doses measured by the ArcCHECK system with and without the plug agreed with the MC simulation to 1.0%. The TPS dose calculation with a 2.5 mm grid overestimated the exit dose by up to 7.2% when the plug was removed. The agreement between the MC and TPS calculations for the ArcCHECK without the plug improved significantly when a 1 mm dose calculation grid was used in the TPS. The noncoplanar delivery test demonstrated that the angular dependency has limited impact on the gamma passing rate (<1.2% drop) for the 2%–3% dose and 2 mm–3 mm DTA criteria. A 1° rotational misalignment introduces 11.3% (3%/3 mm) to 21.3% (1%/1 mm) and 0.2% (3%/3 mm) to 0.8% (1%/1 mm) Gamma passing rate drop for ArcCHECK system and MatriXX system, respectively. Both systems have comparable sensitivity to the AP misalignments. However, a 2 mm RL misalignment introduces gamma passing rate drop ranging from 0.9% (3%/3 mm) to 4.0% (1%/1 mm) and 5.0% (3%/3 mm) to 12.0% (1%/1 mm) for ArcCHECK and MatriXX measurements, respectively. For VMAT plan QA, the gamma analysis passing rates ranged from 96.1% (H&N case) to 99.9% (prostate case), when using the 3%/3 mm DTA criteria for the peripheral dose validation between the TPS and ArcCHCEK measurements. The peripheral dose validation between the MC simulation and ArcCHECK measurements showed at least 97.9% gamma passing rates. The central dose validation also showed an agreement within 2.2% between TPS/MC calculations and ArcCHECK measurements. The worst discrepancy was found in the H&N case, which is the most complex VMAT case. The ArcCHECK system is suitable for VMAT QA evaluation based on the sensitivity to detecting misalignments, the clinical impact of the angular dependency, and the correlation between the dose agreements in the peripheral region and the central region. This work also demonstrated the importance of carrying out a thorough validation of both the TPS and the dosimetry system prior to utilizing it for QA, and the value of having an independent dose calculation tool, such as the MC method, in clinical practice.

PACS number: 87.55.Qr

## I. INTRODUCTION

The successful delivery of volumetric‐modulated arc therapy (VMAT) depends upon controlled and coordinated gantry rotation, dose rate, and accurate MLC leaf positioning. This increased complexity necessitates meticulous quality assurance (QA). Two‐dimensional planar detectors, such as film, diode arrays,^(^ ^1^ ^)^ ion chamber arrays,^(^ ^2^ ^–^ ^3^ ^)^ and electronic portal imaging devices^(^ ^4^ ^)^ are commonly used in QA procedures. Regardless of what type of detector is used, the QA procedure always involves a quantitative comparison between the planned phantom dose and the measured dose. The differences between the two dose distributions are often evaluated using the gamma method^(^ ^5^ ^)^ that takes into account two parameters for every point analyzed in the distribution — a dose value difference and a distance to agreement. Among the benefits of most 2D arrays for QA is the ability to position the detectors in such a way that they will overlap with the location of the treatment target. The QA analysis of the planar dose distribution containing the target area is considered representative of the accuracy of the actual 3D dose delivery.

For 3D dose distributions, several dose planes may be analyzed in order to verify the accuracy of the dose delivery. Gel dosimeters can record the full 3D volumetric dose distribution, but they require MR or CT readout to retrieve the dose values in a postprocessing procedure.^(^ ^6^ ^)^ In order to provide dose measurements that could represent the 3D volumetric dose in a more convenient and less time‐consuming manner, different detector arrangements were proposed. A detector system that uses two diode arrays embedded in a cross‐plane fashion in a cylindrical phantom (Delta4, ScandiDos AB, Uppsala, Sweden) with the ability to perform a time‐resolved dose distribution analysis has been developed.^(^ ^7^ ^)^ The time‐based measurements for rotational therapy can detect both delivery and planning errors since delivery time is correlated with the gantry angle during treatment. In addition, a rotating cylindrical phantom contains an ion chamber array (PTW OCTAVIUS 4D, PTW, Freiburg, Germany) is also proposed to create beam‐to‐detector orthogonal measurements synchronously with the VMAT delivery. A 3D dose distribution can be reconstructed based on the measured plane dose distribution for each gantry angle.^(^ ^8^ ^–^ ^10^ ^)^

A cylindrical diode array detector system with a time‐resolved measurement capability^(^ ^11^ ^)^ was introduced recently for rotational therapy QA procedures. The ArcCHECK system (Sun Nuclear Corp., Melbourne, FL), consisting of 1,386 diode detectors positioned in a cylindrical arrangement, is a three‐dimensional dosimetry system. The hollow core of the dosimetric system introduces less attenuation in the beam path and reduces the total weight for easier setup. A cylindrical detector arrangement ensures orthogonal beam‐to‐detector alignment and, combined with time‐resolved detection, can locate dynamic gantry/leaf motion errors.^(^ ^12^ ^)^ This orthogonal beam‐to‐detector configuration allows the measurement and analysis of the entrance and the exit doses of every single beam direction. These two spatial measurement points correspond to two depths in the beam attenuation curve. Its feasibility for the VMAT QA has been demonstrated by Létourneau et al.^(^ ^11^ ^)^ Feygelman et al.^(^ ^13^ ^)^ further investigated the dose evaluation criteria of this cylindrical dosimetry system with that of the “X” shaped dosimetry system (Delta4) and addressed the fact that a 10% dose cutoff threshold for the gamma test does not have the same meaning for these two dosimetry systems given their different geometry designs.

For general usage of the ArcCHECK system, a 15 cm air gap is present in the cylindrical phantom. This large hollow structure could pose a challenge for the model‐based dose calculation algorithm implemented in the treatment planning system (TPS).^(^ ^14^ ^–^ ^17^ ^)^ To evaluate this issue, we perform an open field dose validation for both the ArcCHECK system and the TPS. Considering the uncertainties of the diode dose measurement and the TPS dose calculation, Monte Carlo (MC) simulation, which has been recognized as the gold standard for estimating the radiotherapy dose measurements/calculations,^(^ ^18^ ^)^ is utilized as an independent reference in this study.

Angular dependency of dose response is among the drawbacks of many radiation detectors.^(^ ^19^ ^)^ The angular response dependency calibration of the ArcCHECK system has been performed by Yan et al.^(^ ^20^ ^)^ However, the diodes are designed and embedded in a nonisotropic structure, and a noncoplanar radiation delivery can exhibit some angular dependency. In this study, we investigated the impact of the angular dependency on the gamma passing rate for the noncoplanar delivery, which has not been evaluated previously.

In contrast to most integrating dosimetry systems (e.g., MapCHECK by Sun Nuclear, MatriXX by IBA dosimetry, Delta4 by ScandiDos AB), which record the dose at the target dose region, the ArcCHECK system records and analyzes the dose at the peripheral dose region. To quantify the advantages of the ArcCHECK system for the VMAT plan QA, we investigated the sensitivity for detecting the delivery defects and compared it with the ion chamber dose measurement in the target and the IBA MatriXX system.

Finally, we investigated the relationship between the peripheral dose agreement and the target dose agreement. We evaluate the dose consistency among ArcCHECK measurements, and TPS and MC dose calculations in the target dose region and in the peripheral dose region for ten clinical VMAT cases.

## II. MATERIALS AND METHODS

The VMAT (RapidArc in this study) treatment plans were designed using the Eclipse system (Version 8.6, Varian Medical System, Palo Alto, CA) with the analytical anisotropic algorithm (AAA) for dose calculations.^(^ ^21^ ^)^ A 21iX linear accelerator equipped with a Millennium MLC incorporating 120 leaves (Varian Medical Systems, Palo Alto, CA) was employed for VMAT plan delivery. The experiments were done with both 6 MV and 10 MV photon beams using real patient plans.

### A. ArcCHECK system and dose calibration

The ArcCHECK diodes are embedded between outer 2.85 cm and 2.90 cm inner acrylic (1.17 g/cc) layers. The diodes are positioned on the surface of a cylinder measuring 21 cm in diameter and form a spiral path of 1 cm pitch and 1 cm interdetector distance. The effective measuring area for each diode is $0.8\times 0.8\;{\text{mm}}^{2}$ and the diode sensitivity is 0.4 nC/Gy.^(^ ^22^ ^)^ The overall phantom has a 26.59 cm outer diameter and a 15.0 cm inner diameter. The 15 cm diameter hollow center is designed to host various accessories for in‐phantom dosimetry purposes. In our study, a homogeneous acrylic plug holding a 0.125 cc ion chamber (Semiflex type, PTW‐Freiburg) was customized to measure the dose at the center of the ArcCHECK phantom. This measurement refers to a point dose in the target region.

The SNC Patient software version 6.1 (Sun Nuclear) was employed for data processing of the ArcCHECK measurements.^(^ ^22^ ^)^ Both the inherent response variations of diode array and the absolute dose response of the ArcCHECK system were calibrated prior to the measurements. The array calibration, which eliminates the slight responses variations of the individual diodes, was performed by the manufacturer and a calibration file was supplied with the system. The absolute dose calibration was performed by the user. The system software compares measured reading of the diodes to a known dose value for a given number of monitor units (MUs) on a calibrated clinical accelerator.

This absolute dose calibration is performed after the TG‐51 linac calibration procedure,^(^ ^23^ ^)^ when the linac is known to deliver 1cGy/MU at $d_{\max}$ for a $10\times 10\;{\text{cm}}^{2}$ field defined at a 100 cm SSD. A 0.125 cc ion chamber is cross‐calibrated using the same setup in the water tank. Afterwards, the ion chamber was placed inside the ArcCHECK plug, enabling dose measurements (100 cm SAD, $10\times 10\;{\text{cm}}^{2}$, 100 MU) at the center of the phantom. The central dose measurements are then used as a baseline for calculating the dose received by the diodes, as required by the system software. The dose received by the diode was determined using the tissue‐maximum ratio (TMR) with effective path lengths (acrylic to water) and inverse square law corrections.^(^ ^20^ ^)^

### B. Monte Carlo simulation software

In this study, a well‐commissioned in‐house Monte Carlo code MCSIM^(^ ^24^ ^)^ was utilized to perform accurate dose calculations. The linear accelerator beam information was included in a source model that was built and commissioned based on measured beam data.^(^ ^25^ ^–^ ^26^ ^)^ The dose calculations were performed in a 3D rectilinear voxel geometry, which was created based on a CT scan of the ArcCHECK phantom. The material and density of each voxel was converted from the CT number based on a piecewise linear conversion curve.

The fluence approximation approach was adopted to efficiently reconstruct the fluence distribution of VMAT delivery by using the DICOM‐RT file that contains the planned delivery parameters: gantry angle, collimator angle, couch angle, field size, and each control point of the MLC sequence. The TPS modeled a full arc treatment with 177 equally‐spaced control points. Each control point contained a static MLC aperture with specific monitor units (MU). Therefore, 176 subfields were defined by the 177 control points and each subfield received an equivalent fluence contribution, according to the gantry rotation range and MLC movement between the adjacent pairs of control points. The VMAT delivery was simulated in a subfield manner, in which the dose for every subfield was calculated sequentially. The final dose of each arc was the sum total of all subfields, and a treatment plan may contain several arcs. The dosimetric features of the MLC, including rounded leaf end transmission, intraleaf transmission, and tongue‐and‐groove effect are also accounted for in the fluence modeling.

### C. Property investigation

#### C.1 Open‐field dose validation

To validate the absolute dose calibration of the ArcCHECK system and also the TPS dose calculations for the ArcCHECK geometry, we compared the absolute depth dose distributions for the $10\times 10\;{\text{cm}}^{2}$ field with two setups: ArcCHECK with a plug (homogeneous geometry) and ArcCHECK without a plug (inhomogeneous geometry). The ArcCHECK system was set at a SAD of 100 cm and 100 MU was delivered for 6 MV and 10 MV photons, respectively.

**Figure 1:**
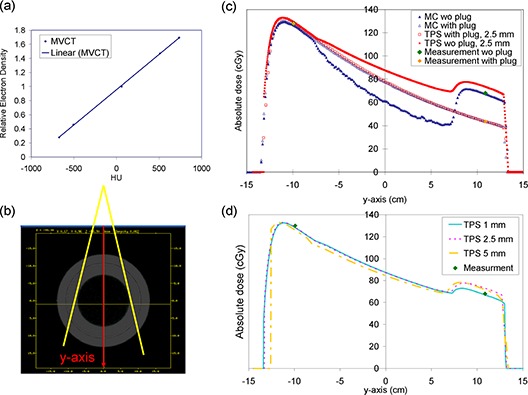
The MVCT ramp (a) provided by the vender. The ArcCHECK geometry (b) reconstructed from the MVCT and the radiation beam orientation. Central beam axis depth dose distributions (c) of the MC and TPS calculations and the ArcCHECK diode measurements for a 10 MV field. The TPS‐calculated depth dose distributions (d) with different calculation grids and the ArcCHECK‐measured doses.

For both the MC simulation and the TPS dose calculation, the MVCT images of the ArcCHECK system and the corresponding CT ramp provided by the vendor were used for the geometry reconstruction to avoid the artifacts from the high atomic number of the diode detectors. The CT ramp is provided in the Fig. 1(a). The resolution of the MC simulation was 2mm×2mm×2.5mm and the statistical uncertainty was within 0.5% (1 σ). The calculated doses were converted to dose to water for the convenience of dose comparisons among measurements, and TPS and MC simulations. The dose calculation grid of the TPS was set at 1mm×1mm,2.5mm×2.5mm, and 5mm×5mm (slice thickness 2.5 mm). For the peripheral dose analysis, which was designed for the ArcCHECK measurement comparisons, the ArcCHECK software reconstructs the peripheral dose distributions from the DICOM‐RT dose files. The MCSIM code records the 3D dose distribution in the ASCII format (3ddose files). To enable the peripheral dose analysis for the MC calculated doses, these 3ddose files were converted to the DICOM‐RT format using the DICOMan^(^ ^27^ ^)^ software (University of Arkansas for Medical Science, Little Rock, AR).

The entrance and the exit doses measured by the ArcCHECK diodes were compared with those calculated by the MC method to evaluate the accuracy of the absolute dose calibrations of the ArcCHECK system. The absolute dose distributions calculated by the TPS were further compared with the MC results to evaluate the accuracy of the TPS dose calculation for the ArcCHECK geometry.

#### C.2 Angular dependency for noncoplanar delivery

To investigate the clinical impact of the angular dependency, the ArcCHECK system was tested with a series of noncoplanar 6 MV beams. The tests were carried out with a $10\times 10\;{\text{cm}}^{2}$ field to eliminate the factors that may affect the results. The photons were delivered laterally to the ArcCHECK system (gantry angle 90°) with those couch angles commonly used in the noncoplanar arc plans (0°, 10°, 20°, 30°, and 40°) to create nonperpendicular irradiation to the diodes. The automatic angular correction function of the ArcCHECK system was switched on during these measurements.

The measured peripheral dose distributions were compared with the one calculated by TPS ones, and the agreements were quantified by the gamma evaluation with various dose (1% to 3%) and distance‐to‐agreement (DTA, 1 mm to 3 mm) criteria. The number of points evaluated in the gamma analysis varies according to the physical size of the dose distribution with respect to the dose threshold value, which is 10% of the maximum dose in this study.

The gamma passing rate of each couch angle was then cross‐compared to evaluate the impact of angular dependency for noncoplanar delivery. For the ArcCHECK system, the gamma passing rates without considering the measurement uncertainty were reported in this study.

#### C.3 Sensitivity of misalignment

To quantify the impact of setup/delivery errors on the gamma passing rate, an arbitrary case (breast) was delivered with various translational (1 mm, 2 mm, 3 mm, 5 mm, 10 mm) and rotational (0° to 3°) misalignments. The phantom translational misalignments were created in anterior‐posterior (AP) and right to left (RL) directions. These misalignments created relative displacements between the treatment beams and the detectors to mimic delivery variation due to positional errors. Measurements were carried out using the ArcCHECK system, MatriXX system, and the ion chamber in the target region. To exclude the effect of the dose gradient inside the ion chamber, we chose measurement points in regions with a low‐dose gradient. To do so, the sensitive volume of the ion chamber was contoured and the difference between the maximum and the minimum dose in the chamber volume was kept within 1%.

The measured doses by the ArcCHECK and the MatriXX were analyzed using the gamma evaluation with the TPS dose calculation as the reference. The gamma passing rates with various criteria (1 mm to 3 mm, 1% to 3%) were compared for the abovementioned misalignment situations. For the ion chamber measurements, the doses measured with misalignments were compared with those measured under the reference conditions.

### D. VMAT plan QA validation

The ability of the ArcCHECK system for VMAT QA was evaluated with ten clinical treatment cases that were planned and delivered for head and neck (H&N), prostate, breast, and abdominal sarcoma patients. A variety of tumor sizes and shapes were selected to represent various clinical scenarios. Table 1 summarizes a description of the dose prescription and VMAT treatment delivery. All of the VMAT plans were delivered in a coplanar manner.

**Table 1:** Treatment plans investigated in this work. Dose prescription and planning control points.

*Treatment Type*	*Prostate 1*	*Head & Neck*	*Breast*	*Prostate 2*	*Prostate 3*	*Prostate 4*	*Pelvis*	*Liver*	*Prostate 5*	*Prostate 6*
Dose prescribed	80Gy/40 fx (95% of PTV covered by Rx)	70Gy/35 fx (95% of PTV covered by Rx)	50Gy/25 fx (85% of PTV covered by Rx)	68Gy/34 fx (95% of PTV covered by Rx)	64Gy/32 fx (95% of PTV covered by Rx)	80Gy/40 fx (95% of PTV covered by Rx)	25Gy/5 fx (100% of PTV covered by Rx)	25Gy/5 fx (99% of PTV covered by Rx)	78Gy/39 fx (95% of PTV covered by Rx)	78Gy/39 fx (95% of PTV covered by Rx)
Planning control points	ARC I 177 CP 10X; ARC II 129 CP 10X	ARC I 177 CP 6X ARC II‐III 97 CP 6X	ARC I‐II‐III 129 CP 6X	ARC I‐II 177 CP 10X	ARC I‐II 177 CP 10X	ARC I‐II 177 CP 10X	ARC I 177 CP 10X	ARC I 177 CP 10X ARC II 177 CP 10X	ARC I‐II 177 CP 10X	ARC I 177 CP 10X ARC II 129 CP 10X

Two ArcCHECK measurements were carried out for each case. First, the peripheral dose distributions were measured using the integrating mode without the plug, which is the original design of the ArcCHECK system. In addition, the central dose of each plan was measured with the acrylic plug holding a 0.125 cc ion chamber. The VMAT plans were calculated using both TPS and the MC method with the corresponding MV CT scans of the ArcCHECK system with and without the plug inside. For the ion chamber dose estimation, the sensitive volume of the ion chamber was contoured and the average dose of the sensitive volume was scored in both MC and TPS calculations.

For the central dose comparisons, the absolute dose differences among the ion chamber measurements and the MC and TPS calculations were evaluated. The peripheral dose distributions were evaluated with the Gamma criterion of 3% dose and 3mm DTA.

## III. RESULTS

### A. Property investigation

#### A.1 Open‐field dose validation

Figure 1(b) shows the ArcCHECK geometry reconstructed from the MVCT and the beam orientation. The results of the 6 MV and 10 MV photon beams demonstrate a similar trend. Here we only show the 10 MV results. The absolute depth dose distributions of the 10 MV photon beam are shown in Fig. 1(c). The ArcCHECK‐measured entrance and exit doses agree with the MC calculations for both ArcCHECK geometries with and without the plug. The deviations between the ArcCHECK‐measured and the MC‐calculated doses are within 1.0% for the entrance dose and 0.7% for the exit dose without the plug. For the ArcCHECK with the plug, the difference between the ArcCHECK‐measured and the MC‐calculated exit doses is within 0.2%. These agreements indicate that the ArcCHECK has been properly calibrated for the absolute dose measurements.

A common mesh grid for the TPS calculation is 2.5 mm, which is a compromise between dose calculation accuracy and calculation time. We first compare the TPS calculations with a 2.5 mm grid size with the MC calculations. As seen in Fig. 1(c), the depth dose distribution calculated by the TPS (hollow red squares) is consistent with the one calculated by the MC method (hollow blue triangles) under the homogeneous geometry (ArcCHECK with the plug). The differences are observed in the exit doses when the acrylic plug was absent. For the 10 MV photons, the ArcCHECK‐measured (green diamonds) and the MC‐calculated (solid blue triangles) exit doses agree with each other. But, the TPS (solid red squares) overestimates the exit dose by 7.2% in absence of the plug in the phantom geometry.

To further investigate this phenomenon, we calculated the open‐field doses with the following grid sizes: 1 mm, 2.5 mm, and 5 mm. As shown in Fig. 1(d), the differences between the measured and the calculated exit doses decreases with the grid size. The exit dose calculated with 1 mm grid showed a good agreement with measurements and; hence, the 1 mm calculation grid was utilized in this study to calculate the verification plans for the ArcCHECK system without the plug.

#### A.2 Angular dependency for noncoplanar delivery

**Figure 2:**
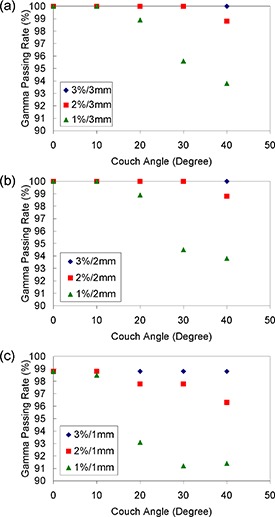
The gamma passing rates for various gamma criteria and couch angles.

Figure 2 shows the gamma passing rates with respect to the various rotation angles and the nine gamma analysis criteria varying from 1% to 3% dose and 1 to 3 mm DTA. As seen in Fig. 2(a), with the 3%/3 mm criteria that are commonly used in the IMRT QA, the noncoplanar delivery has no impact on the gamma passing rate (100% passing rate for all couch angles). As the dose difference criteria get stricter and the couch angle increased, the gamma passing rates of 2%/3 mm and 1%/3 mm criteria drop from 100% to 98.8% and to 93.8%, respectively, when the couch angle rotates from 0° to 40°. However, when the couch angle is less than 20°, the gamma passing rates for the 3%/3 mm and 2%/3 mm criteria remain at 100%, and the gamma passing rate for the 1%/3 mm criterion decreases from 100% to 98.9%.

Similar trends are seen when we reduce the DTA criterion to 2 mm (Fig. 2(b)). For the 1 mm DTA criterion, the impact of angular dependency is more pronounced (Fig. 2(b)). The gamma passing rates for 2%/1 mm and 1%/1 mm criteria are reduced to 97.5% and 92.6%, respectively, when the couch angle is 40°. However, when the 3% dose criterion is used, the gamma passing rates under couch angle 40° remain the same as that for couch angle 0°.

#### A.3 Sensitivity of misalignment

**Figure 3:**
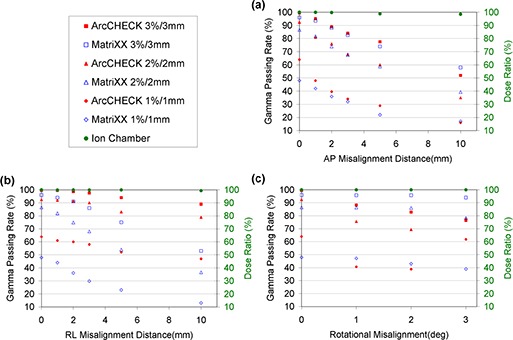
The gamma passing rates of ArcCHECK and MatriXX measurements and the ratios of central doses measured by an ion chamber with respect to: (a) anterior‐to‐posterior misalignments, (b) left‐to‐right misalignments, (c) rotational misalignments.

Figure 3 describes the sensitivity for detecting the translational misalignments for the ArCHECK, MatriXX, and the ion chamber measurements. For the point dose measurements, the results are represented as the dose ratio between the measured dose and the reference condition. It is evident that the translational misalignments have limited impact on the measured point dose. For the misalignment conditions tested in this study, the maximum central dose variation is 1.5%.

As seen in Fig. 3(a), the ArcCHECK and the MatriXX show similar sensitivity to AP misalignments. For a 1 cm AP misalignment, the gamma passing rates for the ArcCHECK measurements decrease by 47.5% for 3%/3 mm and by 57.5% for 2%/2 mm criteria. In contrast, the reduction of the gamma passing rate for MatriXX with a 2 mm AP misalignment ranges from 38.0% (3%/3 mm) to 47.2% (2%/2 mm). Furthermore, the ArcCHECK appears to be more sensitive to the small AP directional misalignment (< 2 mm). For the ArcCHECK, the gamma passing rate drops from 99.5% to 89.1% versus 96% to 88% for the MatriXX when a 2 mm misalignment is applied in the AP direction. For the lateral (RL) misalignment, the MatriXX is significantly more sensitive than the ArcCHECK, as demonstrated in Fig. 3(b). A 2 mm RL misalignment induces a gamma passing rate drop ranging from 0.9% (3%/3 mm) to 4.0% (1%/1 mm) and 5.0% (3%/3 mm) to 12.0% (1%/1 mm) for ArcCHECK and MatriXX measurements, respectively.

Figure 3(c) shows the sensitivity of detecting the rotational misalignments. Again, point dose measurements can hardly reflect the misalignment in the measured dose. MatriXX shows a limited ability of detecting the rotational misalignments. For the 3%/3 mm criterion, the gamma passing rate decreases from 96% to 93.8% when misalignment of 3° is applied. The sensitivity is slightly improved as the evaluation criterion gets stricter; the gamma passing rate drops from 48% to 39.2% for the1%/1 mm criterion. In contrast, the ArcCHECK system is more sensitive to the rotational misalignment. A 1° misalignment results in a reduction of 11.3% (3%/3 mm) and 21.3% (1%/1 mm) in the gamma passing rate, respectively.

### B. VMAT plan QA validation

Table 2 shows the peripheral dose comparison between ArcCHECK measurements and TPS/MC calculations. An additional comparison is carried out between the TPS calculations and the MC simulations. The gamma evaluation between the MC‐calculated and the ArcCHECK‐measured peripheral doses show high passing rates which are above 97.9% for all cases. With a 1 mm calculation grid, the doses calculated by the TPS achieve at least 96.1% passing rate when comparing with the ArcCHECK measurements. For all prostate cases, the passing rate varies from 96.5% to 99.8% for the 3%/3 mm tolerance. The H&N case has a 96.1% passing rate, and the passing rate for the breast, liver, and pelvic cases was found to be 99.5%, 99.8%, and 99.4%, respectively, for 3%/3 mm test. The comparison between the MC simulation and the TPS also demonstrate a reasonable passing rate (>96.7%).

**Table 2:** Gamma passing rates (3%/3 mm) of VMAT treatment plans based on the peripheral dose distributions.

*Treatment Type*	*Meas.‐TPS Passing Rate (%)*	*Meas.‐MC Passing Rate (%)*	*TPS – MC Passing Rate (%)*
Prostate 1	99.3	98.6	99.9
HN	96.1	97.9	96.7
Breast	99.5	97.9	98.1
Prostate 2	97.7	99.9	96.8
Prostate 3	96.5	99.6	97.5
Prostate 4	99.7	99.7	~100
Liver	98.6	99.9	99.9
Pelvis	99.4	99.6	99.6
Prostate 5	99.9	99.8	~100
Prostate 6	99.6	99.4	99.5

Table 3 shows the central axis phantom dose measurements in cGy, along with the dose calculated by both the TPS and the MC method. The percentage differences between the calculations and the measurements are also listed. For the prostate cases, the measured doses agreed with MC and TPS within 0.8% and 1.5%, respectively. The differences between the TPS calculation and the measurement for the breast, liver, and pelvic cases are found to be 0.3%, 0.8%, and 2.2%, respectively, while the MC simulations show less than 1.3% dose differences comparing with measurement for those cases. The largest differences are found in the H&N case, which is the most complex VMAT plan; the TPS and the MC calculations show a 1.5% and 1.4% differences from the measured dose.

**Table 3:** Dose values measured by ion chamber and calculated by TPS and MC at the center of the ArcCHECK phantom for the selected plans.

*Treatment Type*	*Measurement (cGy)*	*TPS (cGy)*	*MC (cGy)*	*TPS to Meas. (percent)*	*MC to Meas. (percent)*
Prostate 1	215.7	216.2	215.4	0.2	−0.1
HN	108.6	110.2	110.1	1.5	1.4
Breast	152.8	153.3	150.8	0.3	−1.3
Prostate 2	213.8	211.5	215.3	−1.1	0.7
Prostate 3	216.1	219.3	214.4	1.5	−0.8
Prostate 4	236.2	234.3	234.6	−0.8	−0.7
Liver	565.1	569.6	564.6	0.8	−0.1
Pelvis	504.4	515.6	503.6	2.2	0.2
Prostate 5	232.3	231.0	230.5	−0.6	−0.7
Prostate 6	222.5	220.6	222.1	−0.6	−0.2

## IV. DISCUSSION & CONCLUSION

The ArcCHECK system is a novel detector system for radiation dosimetry. The system uses a cylindrical diode array that ensures an orthogonal beam‐to‐diode configuration for all beam angles. For a coplanar treatment delivery, the ArcCHECK system accumulates and records the dose in two areas on the rectangular dose distribution, one for the diodes close to the beam source, and the other for the diodes measuring the beam exiting the phantom. Inherent to the system is an assumption that central (target) dose is supposed to agree with the planed one if both the entrance and the exit doses agree with the predicted values. This assumption is certainly true, if we take the concept of the *in‐vivo* dosimetry^(^ ^28^ ^–^ ^30^ ^)^ — the midline dose of one single beam can be derived from either the geometric or the arithmetic mean of the entrance and the exit doses in the symmetrical homogeneous geometry since the photon attenuation along the depth can be described as an exponential function. Therefore, the ArcCHECK‐measured peripheral dose, which is the summation of the entrance and the exit doses, is compared to the one calculated by the TPS. In this study, we utilized the MC method to evaluate both the peripheral doses and the central doses for ten coplanar VMAT plans. For the peripheral doses evaluations, the gamma passing rates exceed 96.1%. The average gamma passing rate of the selected cases is 98.6% and is comparable to the average gamma passing rate (98.4% for the 2° control point increment of SmartArc plans) reported by Feygelman et al.^(^ ^13^ ^)^ AAPM TG‐119^(^ ^31^ ^)^ suggests the value for points passing gamma criteria of 3%/3 mm be set to 88%–90%. This demonstrates that ArcCHECK system is suitable for VMAT plan QA. In addition, these peripheral dose agreements also reflect the agreements in the target dose, as demonstrated in the central dose validation (Table 2). Taking the H&N case, which is the most complex VMAT case, as an example, the worse peripheral dose agreement (96.1%) translated into a worse agreement in the target dose.

The Eclipse TPS used in this study employs an anisotropic analytical algorithm (AAA)^(^ ^32^ ^)^ to calculate the dose more precisely at low density regions.^(^ ^33^ ^)^ The AAA algorithm makes use of precalculated Monte Carlo dose kernels and provides better dose modeling than the pencil beam algorithm, especially for heterogeneous media.^(^ ^34^ ^)^ However, in the open‐field validation, we found that the 15 cm hollow structure in the ArcCHECK system indeed leads to an overestimation in the TPS dose calculation (Fig. 1). The exit dose overestimation phenomenon is not only seen in the open‐field validation, but also is observed in the VMAT plan validation. For instance, the passing rate is found to be only 80.9% for the breast case when 2.5 mm calculation grid is utilized in the TPS and evaluated against the MC calculated doses. It increased to 98.1% when the calculation grid was set to 1 mm. Although the ability of AAA algorithm for handling the inhomogeneity in the patient body has been proven by many investigators, the 15 cm hollow gap in the ArcCHECK system is relatively larger than the inhomogeneities found in the human body. As indicated by Gray et al.,^(^ ^16^ ^)^ the dose beyond a large air gap was reduced due to a decrease in scattered radiation reaching the point of interest and hence an overestimation is observed in the TPS dose calculation. This issue was also mentioned by Feygelman et al.^(^ ^13^ ^)^ for a different TPS (Pinnacle v. 9.0, Philips Radiation Oncology Systems, Fitchburg, WI). Further investigation regarding this phenomenon would be favorable. However, this finding demonstrates the necessity to carry out a thorough validation of both detection and TPS before introducing any new system in the clinic. An accurate, independent algorithm, such as the MC method, would be valuable for the validation among different dosimetry systems. The other practical solution is to operate the ArcCHECK system with the plug inside, since the cavity would introduce errors to the TPS dose calculations and the TPS dose calculations with 1 mm grid size would take more than an hour CPU time for a typical VMAT plan.

The feature of having two detector locations monitoring a given beam enables the correction for the angular dependency. Yan et al.^(^ ^20^ ^)^ reported that the angular dependency can lead to an up to 8% difference in the diode response. For the noncoplanar delivery test proceed in this study, we notice that the impact of angular dependency on the gamma passing rate is more pronounced on the dose criteria than the DTA criteria (Figs. 2(a)) and (2c). This is reasonable because the angular dependency affects the dose response of the diodes. With the automatic angular dependency correction provided by the ArcCHECK, the impact on the gamma passing rate is found to be insignificant, especially when the couch angle is less than 20°. Although a larger drop of the gamma passing rate is seen in the 1%/1 mm evaluation, this criterion is too strict for the VMAT QA (mostly 2% to 3% dose and 2 to 3 mm DTA). For the 2%/2 mm criterion, the deviation of the gamma evaluation is 1.2% when the couch angle is 40°. We can therefore conclude that the impact of angular dependency for noncoplanar delivery is negligible.

In the misalignment tests, we have demonstrated that the conventional point dose verifications are not sensitive to either translational or rotational misalignments (Fig. 3). The point dose measurements highly depend on the dose gradient inside the target. The breast case tested in this study is categorized as big tumor, which yields a homogeneous dose distribution inside the target. In contrast, both the ArcCHECK and the MatriXX exhibit higher sensitivity to the misalignments. The difference of the misalignment sensitivity between these two systems is a result of the different design of the detector arrangements. With the cylindrical geometry and the spiral diode distribution, the ArcCHECK has higher ability to identify the rotational misalignments than the MatriXX. This is because the translational shift caused by the rotational misalignment is larger in the peripheral area than the center area. On the other hand, the translational misalignment tests indicate the two systems have comparable sensitivity to the AP misalignment, but the MatriXX system is more sensitive to the RL shift. This is because the RL shift in the center would introduce an absolute shift to all the detectors in the MatriXX. For the ArcCHECK, the RL shift in the center would translate into a smaller absolute shift to the cylindrical detector plane. In a similar test carried out by Létourneau et al.,^(^ ^11^ ^)^ the ArcCHECK also showed a slightly lower sensitivity to the RL misalignments than the AP misalignments. In Létourneau's work, the authors compared the misalignment sensitivity for H&N, sarcoma, and prostate cases and stated that the sensitivity could be case‐dependent. In this study, we demonstrate the misalignment sensitivity of the ArcCHECK system for the breast case. This study provides a qualitative comparison of the misalignment sensitivity between the ArcCHECK and the MatriXX systems. However, a quantitative comparison between these two systems requires more cases on different treatment sites, which is beyond the scope of the current study.

**Figure 4:**
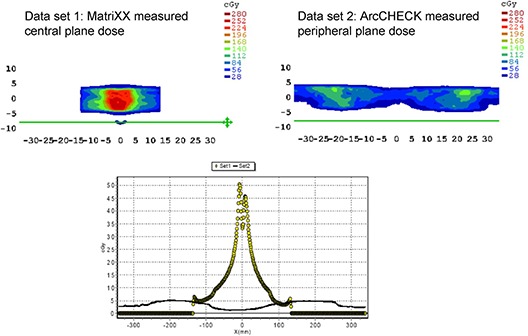
Two dose distributions for a prostate treatment: top left, the central plane dose measured by the MatriXX; top right, the peripheral plane dose measured by the ArcCHECK. Bottom: X‐axis dose profile for both plane distributions; the green lines in the MatriXX‐ and ArcCHECK‐measured dose distributions indicate an undesired normal tissue dose for the coronal slice which is difficult to detect for the cylindrical plane distribution. (Taken from the MapCHECK software.)

The other difference between the planar dose verification in the high‐dose region and the peripheral region is that the peripheral dose is supposed to acquire more information because the entrance and the exit doses of each beam are detected by the cylindrical diode array. However, as seen in Fig. 4, there is an unexpected dose (5 to 50 cGy) in the normal tissue area shown in the dose distribution measured by the MatriXX but not the ArcCHECK. This is a VMAT plan for a prostate treatment and the unexpected dose is introduced by leaf abutment inside the fixed jaw locations. This kind of unexpected dose can be observed by the MatriXX only when a correct dose plane is chosen for the measurement. Theoretically, the ArcCHECK system should be able to detect this unexpected dose in any situation. However, owing to the cutoff value preset by the vendor, the ArcCHECK system does not display the doses below 10% of the maximum dose. A recommendation is made to the vendor for allowing the users to set their own cutoff value. Additionally, the users have to pay attention to the lower dose area because it could contribute to certain amount of dose anywhere inside the geometry. This finding also reveals that the dose distributions measured by the dosimetry systems with different geometry designs can be used to validate different aspects of a treatment plan. The evaluation criterion should be established based on the properties of the dosimetry system.

## ACKNOWLEDGMENTS

The authors would like to thank Sun Nuclear, Corp. for technical support and Dr. Yulong Yan for lending his support to the DICOMan software.

## Supporting information

Supplementary Material

Supplementary Material

Supplementary Material

Supplementary Material

Supplementary Material

Supplementary Material
