# Supplementary figures and images for: Measurement comparison and Monte Carlo analysis for volumetric‐modulated arc therapy (VMAT) delivery verification using the ArcCHECK dosimetry system
Source: J Appl Clin Med Phys. 2013 May 6;14(2):220–33. doi: 10.1120/jacmp.v14i2.3929 (PMC5714369; doi:10.1120/jacmp.v14i2.3929)

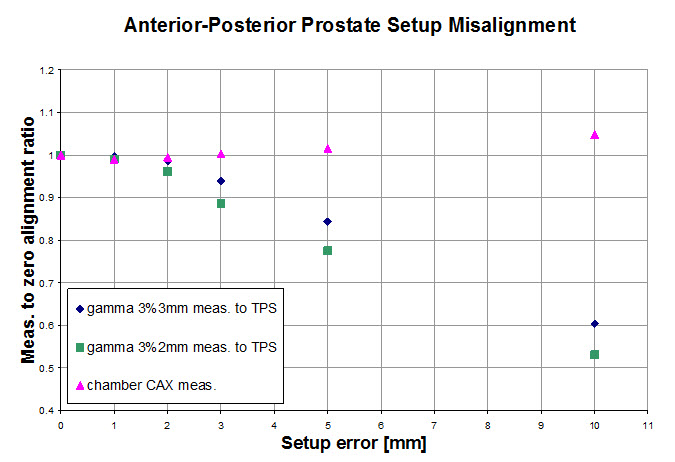

Supplement: Supplementary file 1 — Supplementary Material [file ACM2-14-220-s001.jpg]

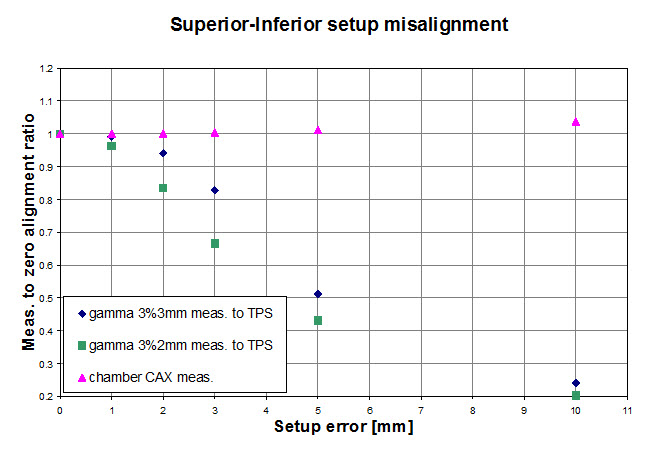

Supplement: Supplementary file 2 — Supplementary Material [file ACM2-14-220-s002.jpg]

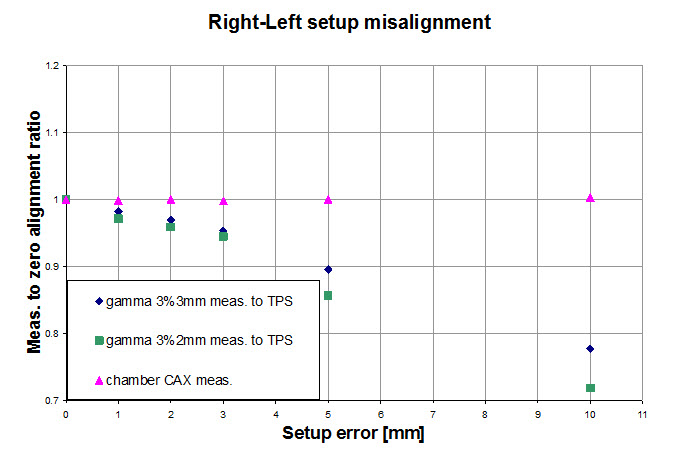

Supplement: Supplementary file 3 — Supplementary Material [file ACM2-14-220-s003.jpg]

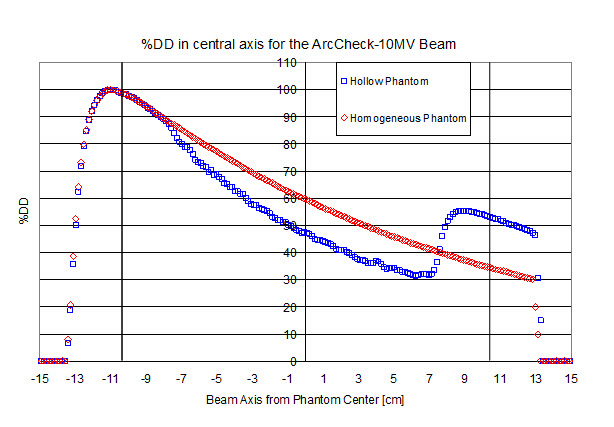

Supplement: Supplementary file 4 — Supplementary Material [file ACM2-14-220-s004.jpg]

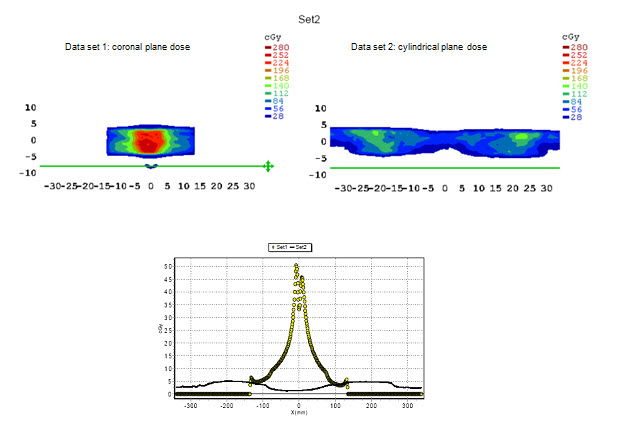

Supplement: Supplementary file 5 — Supplementary Material [file ACM2-14-220-s005.jpg]

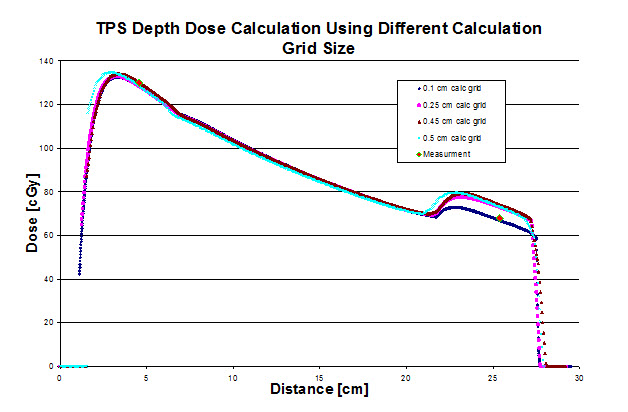

Supplement: Supplementary file 6 — Supplementary Material [file ACM2-14-220-s006.jpg]
